# EGFR Expression in HER2-Driven Breast Cancer Cells

**DOI:** 10.3390/ijms21239008

**Published:** 2020-11-27

**Authors:** Florian Weinberg, Diana B. Peckys, Niels de Jonge

**Affiliations:** 1 INM—Leibniz Institute for New Materials, 66123 Saarbrücken, Germany; florian.weinberg@leibniz-inm.de; 2 Department of Biophysics, Saarland University, 66421 Homburg, Germany; diana.peckys@uks.eu; 3 Department of Physics, Saarland University, 66123 Saarbrücken, Germany

**Keywords:** EGFR, HER2, electron microscopy, single molecule, correlative microscopy, dual labeling

## Abstract

The epidermal growth factor receptor HER2 is overexpressed in 20% of breast cancer cases. HER2 is an orphan receptor that is activated ligand-independently by homodimerization. In addition, HER2 is able to heterodimerize with EGFR, HER3, and HER4. Heterodimerization has been proposed as a mechanism of resistance to therapy for HER2 overexpressing breast cancer. Here, a method is presented for the simultaneous detection of individual EGFR and HER2 receptors in the plasma membrane of breast cancer cells via specific labeling with quantum dot nanoparticles (QDs). Correlative fluorescence microscopy and liquid phase electron microscopy were used to analyze the plasma membrane expression levels of both receptors in individual intact cells. Fluorescent single-cell analysis of SKBR3 breast cancer cells dual-labeled for EGFR and HER2 revealed a heterogeneous expression for receptors within both the cell population as well as within individual cells. Subsequent electron microscopy of individual cells allowed the determination of individual receptors label distributions. QD-labeled EGFR was observed with a surface density of (0.5–5) × 10^1^ QDs/µm^2^, whereas labeled HER2 expression was higher ranging from (2–10) × 10^2^ QDs/µm^2^. Although most SKBR3 cells expressed low levels of EGFR, an enrichment was observed at large plasma membrane protrusions, and amongst a newly discovered cellular subpopulation termed EGFR-enriched cells.

## 1. Introduction

The tyrosine kinase human epidermal growth factor receptor 2 (HER2, also ErbB2) belongs to the epidermal growth factor receptor (EGFR) family, consisting of EGFR, HER2, HER3 and HER4 [1]. HER2 represents an overexpressed oncoprotein responsible for approximately 20% of all breast cancer cases [2,3]. HER2 signaling promotes cell survival and cell growth, and is initiated by receptor dimerization [4]. Dimerization occurs ligand-independent in the case of HER2 homodimers, or ligand-dependent in heterodimers with EGFR, HER3 and HER4 [5]. Dimerization of receptors leads to the activation and phosphorylation of the carboxyl tail of the “activator” kinase. This will in turn phosphorylate and activate the “receiver” kinase of the dimerization partner, leading to full activation of downstream signaling pathways. Preventing dimerization of kinases and inhibiting their kinase activity is a therapeutic strategy to block HER2 signaling in breast cancer [3]. When combined with chemotherapy, current therapeutic strategies for HER2 driven breast cancer include I) blocking receptor dimerization with antibodies, such as trastuzumab and pertuzumab, designed to bind to the extracellular domain of HER2 or II) blocking the kinase activity of the receptors with small molecule compounds like lapatinib and neratinib [6,7,8,9]. In addition, antibody drug conjugates like trastuzumab-emtansine (T-DM1) are used to specifically kill HER2 positive cancer cells with the conjugated cytotoxic drug emtansine (DM1), while bispecific antibodies are used to attract and activate components of the immune system against HER2 positive tumor cells [10].

EGFR is, besides HER3, the preferred heterodimerization partner of HER2 [5], but its role in HER2-overexpressing cancer is still not fully understood [11,12]. Elevated EGFR expression is observed in 54% of basal-type breast cancers [13], an aggressive type of triple negative breast cancer associated with poor survival [14]. In SKBR3 cells, an immortalized breast cancer cell line serving as a common model for HER2 driven breast cancer, HER2 expression is 20-fold higher in average compared to EGFR [15,16]. In vitro studies in this cell line reported therapy resistance development to trastuzumab upon EGFR overexpression [17]. HER4 is a further candidate for forming heterodimers with other members of the HER family but the role HER4 plays in the course of breast cancer has long been unclear [18,19]. 

In addition to breast cancer, HER2 overexpression exists in many other cancer types, for instance in bladder cancer, where co-overexpression of HER2 and EGFR was reported for the majority of the tumors and metastases [20]. In prostate cancer overexpression of HER2 and EGFR is associated with cancer progression and bone metastasis [21]. These findings underline the necessity to develop labeling protocols allowing the investigation of single cells at the single-protein level. Particularly, the presence of less abundant cell subtypes within a tumor, for example, cancer stem cells, might be missed with quantification methods only determining the average level of the expression of a gene or protein of interest, which is dominated by the bulk (most abundant) type of cells. Although these subpopulations of cells account only for a minimal proportion of the whole tumor, their high plasticity provides a source of tumor renewal, giving rise to several types of cancer cells within the same tumor that drastically differ in their response to therapy [22,23,24,25,26]. 

Quantitative approaches to determine the expression of EGFR, HER2, and HER3 in patient samples and cell lines have been published using a variety of techniques including super resolution microscopy, Förster resonance energy transfer (FRET), proximity ligation assay, DNA aptamer ligation assay, and genetic protein tags [27,28,29,30,31,32,33]. However, these methods either require (artificial) exogenous protein expression in HER2 non-expressing cell lines, are incapable of sufficiently resolving single protein distribution, require secondary signal amplification leading to steric hindrance and thus insufficient labeling, or provide false positive signals at the higher receptor densities found in cancer cells [34,35]. 

Here, we present a dual-labeling approach for correlative light microscopy and liquid phase electron microscopy for endogenous HER2 and EGFR receptors within the plasma membranes of intact SKBR3 cells. This cell line was chosen as it widely serves as a model for HER2-driven breast cancer. EGFR and HER2 receptor labeling served as a proof of principle approach for two distinct proteins. The advantages of electron microscopy and fluorescent immunolabeling were combined to achieve single protein resolution along with cell subtype identification and subsequent single cell analysis. To this end, EGFR and HER2 receptors were labeled on the surface of fixed SKBR3 cells with fluorescent quantum dot nanoparticles (QDs) of different sizes and fluorescent wavelengths. The fixation method was optimized to preserve membrane structures like large membrane protrusion, ruffles, local microclusters of receptors and to prevent fixation induced labeling artifacts [31,34,36]. Cells of various phenotypes were observed within the SKBR3 cell line. Most cells expressed HER2 with a heterogeneous distribution of local molecular densities with HER2 receptors appearing in clustered regions of elongated shapes that are associated with cell membrane deformation [37], whereas some cells were characterized by large membrane protrusions densely packed with HER2 molecules. In these latter regions, EGFR receptors were also expressed with a higher average density. Furthermore, a small subgroup of SKBR3 cells were identified and termed EGFR-enriched cells that were characterized by elevated levels of EGFR expression a factor of two to three largen than that of the bulk majority of cells. Electron microscopy enabled the number of receptors to be determined for different cell types and membrane regions.

## 2. Results

### 2.1. Establishment of a Dual-Labeling Approach for EGFR and HER2 for Correlative Light and Electron Microscopy

A dual-labeling approach was established to simultaneously analyze the expression of individual EGFR and HER2 receptors on the surface of SKBR3 cells with correlative light and electron microscopy. To this end, our previously described two-step HER2 specific labeling protocol was extended, modified, and optimized [34]. Briefly, SKBR3 cells were grown on electron microscope-suited microchips, starved to prevent receptor internalization or recycling, and fixed. 

**Figure 1:**
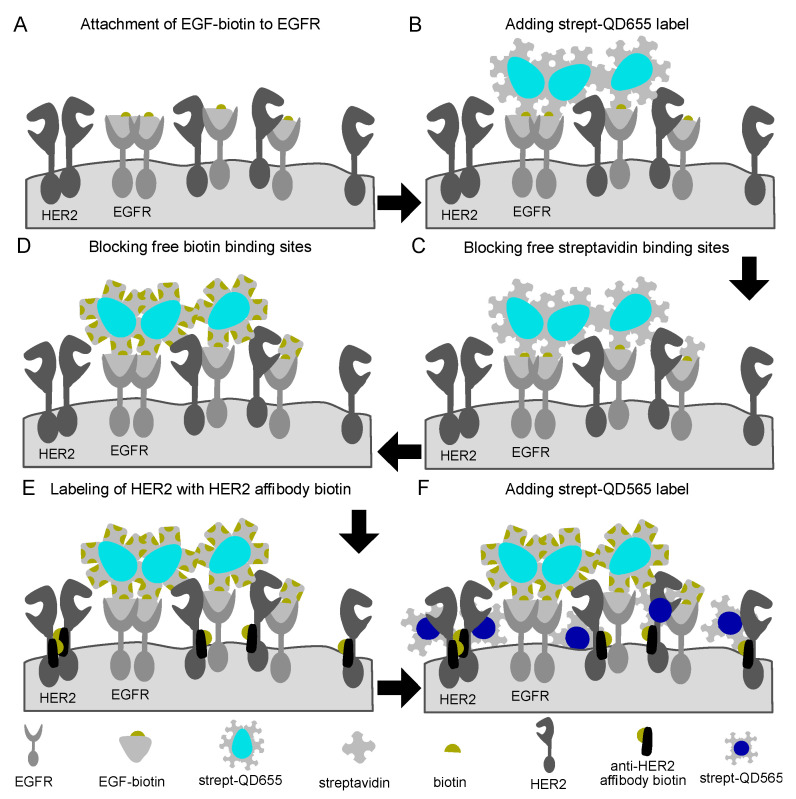
Schematic representation of the labeling procedure for epidermal growth factor receptor (EGFR) and human epidermal growth factor receptor 2 (HER2) receptors in fixed cancer cells. (**A**) First, epidermal growth factor (EGF) with a single conjugated biotin molecule was incubated after which (**B**) streptavidin conjugated quantum dots 655 (streptQD655) were attached. (**C**) Intermediated blocking steps with free streptavidin and (**D**) biotin solution were applied to saturate any remaining free biotin or streptavidin binding sides from the EGFR-QD655 label. For HER2 labeling, (**E**) first, an HER2 specific mono-biotin conjugated Affibody was attached to the extracellular domain IV of HER2, then (**F**) streptQD565 was attached.

Several different double labeling strategies for EGFR and HER2 were tested, including one-step labeling, using gold nanoparticles instead of QDs, and comparing sequential label assembly on the fixed or living cells (see Supplemental Figures S1–S3). The successful protocol was based on 2-step labeling protocols for each receptor, using biotin-streptavidin binding reactions, including intermediate blocking steps, and use of QDs of two different sizes (Figure 1). First, EGFR receptors were labeled by incubation with EGF modified with a single biotin moiety, followed by addition of streptavidin conjugated, fluorescent and electron-dense nanoparticles (streptQD655). This resulted in a maximum 1:1:1 labeling ratio for EGFR:EGFbiotin:streptQD655 (EGFR-QD655). Thereafter, any remaining free streptavidin- and biotin binding sites of the EGFR-QD655 probes, and on the cell surfaces were saturated by incubation with concentration-optimized free streptavidin and biotin solutions, respectively (Supplemental Figure S4). Finally, HER2 was labeled similarly to EGFR, first by attachment of an anti-HER2 specific Affibody carrying a single biotin, then by addition of streptavidin conjugated nanoparticles of a different size (streptQD565). This also resulted in a maximum 1:1:1 labeling ratio for HER2:anti-HER2 Affibody biotin:streptQD565 (HER2-QD565). 

The optimized protocol consisted of a pre-fixation step with 3% formaldehyde for five minutes, followed by a fixation step of 3% formaldehyde mixed with 0.2% glutaraldehyde for five minutes. This combination of fixatives and the subsequent fixation order prevented formation of autofluorescence caused by glutaraldehyde (Supplemental Figure S5A). However, we observed that the presence of glutaraldehyde caused a certain degree of “stickiness” of surfaces (substrate and cells) after fixation, probably caused by the formation of instable Schiff bases and activated ϵ-amino group of lysine residues on the surfaces of proteins [38] leading to non-specific binding of the anti-HER2 Affibody (Supplemental Figure S5B). The non-specific capture of label reagent was reduced by quenching with the amine group containing protein solution glycine (1 M concentration) and by the two-step fixation procedure (Supplemental Figure S5B). Also, by using this two-step fixation protocol, membranous structures like LMPs, microclusters of receptors (clustered regions), and flattened areas were conserved. 

Our previously established protocol [34] involved incubation of the Affibody on living cells but pre-fixation was needed here. To ensure that the binding of the anti-HER2 Affibody would not be impaired by fixation, the binding of the Affibody (fluorescein isothiocyanate (FITC)-conjugated) to living cells was compared with the binding to fixed cells via fluorescence microscopy, confirming no impairment after cell fixation (Supplemental Figure S6). 

The fluorescent and electron dense QDs are highly resistant to bleaching and superior to conventional fluorophores in terms of brightness [39,40]. The emitted fluorescence is dependent on the particle size; streptQD655 has a diameter of 14–18 nm and emits red fluorescence, whereas streptQD565 has a diameter of 12 nm and emits yellow fluorescence [41]. After staining, the cells were analyzed with fluorescence microscopy using QD-optimized filter sets to allow for a precise separation of the two labels. The non-specific binding of streptQDs to fixed cell surfaces and substrates was examined (Supplemental Figure S7), though this proved to be negligible. Finally, after fluorescence imaging, the cells were post-fixed with 2% glutaraldehyde and stored until electron microscopy analysis.

### 2.2. Growth Factor Expression Differs on Individual SKBR3 Cells

**Figure 2:**
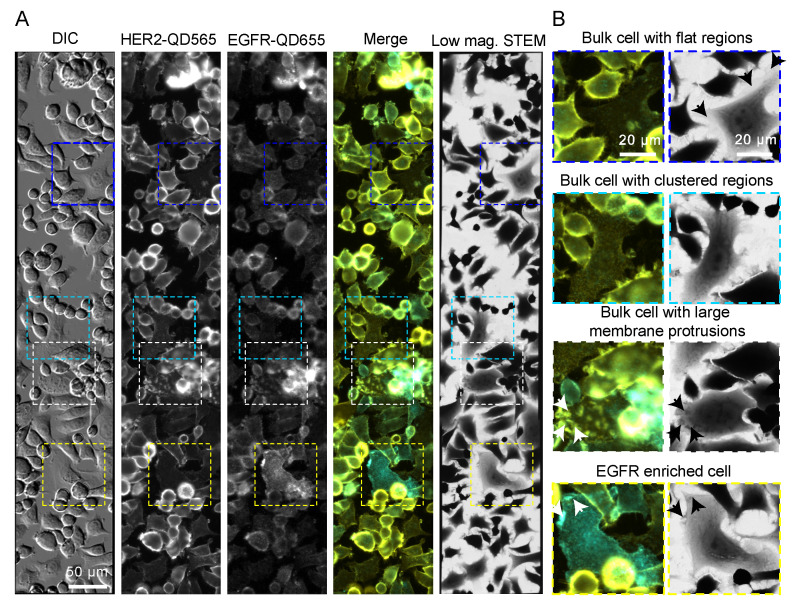
EGFR and HER2 dual labeling on SKBR3 cells. (**A**) Correlative fluorescence microscopy and low magnification bright field scanning transmission electron microscopy (STEM) of SKBR3 cells labeled for HER2-QD565 and EGFR-QD655. Light microscope images were acquired with a 20× objective and automatically stitched together. The same region is shown in the STEM image at a magnification (M) = 800×. Colored rectangles indicate the magnified areas depicted in (**B**) highlighting the identified cell surface regions and subtypes of cells for SKBR3: a bulk cell with flat appearing regions (blue frame, arrows), a bulk cell with clustered HER2 expression distributed over the surface of the cells (cyan frame), a bulk cell with large membrane protrusions (arrows), dorsal and lateral ruffles (white frame), and an EGFR enriched cell with clustered regions and large membrane protrusions (arrows) (yellow frame). Colors in the merged images: yellow for HER2 and cyan for EGFR. Scale bars: 50 µm (**A**) and 20 µm (**B**).

SKBR3 cells, a model for HER2 positive breast cancer, express low levels of EGFR based on RNA analysis [42]. Indeed, fluorescence analysis of SKBR3 cells displayed a rather low expression of EGFR as judged by fluorescence intensity, especially when compared to the HER2 expression (Figure 2). Different expression phenotypes were observed for individual cells and for different cell surface areas on individual cells (Figure 2B). For most cells, termed bulk from here on, HER2 expression was enriched on the cell surface in clustered stripes or patches (see bulk cell with clustered regions in Figure 2B and Supplemental Movie S1). Strong enrichments in HER2 expression were observed for large membrane protrusions (LMPs), like dorsal and lateral ruffles as compared to the clustered regions [31] (see bulk cell with LMPs in Figure 2B and Supplemental Movie S1). Surface areas of cells appearing flat in the differential interference contrast (DIC) channel were characterized by a rather homogenous HER2 expression pattern with no local clusters or enrichments (see bulk cell with flat region in Figure 2B and Supplemental Movies S2 and S3). Bulk cells expressed low levels of EGFR homogenously on the cell surface, with increased EGFR expression only notable at LMPs. In addition, a few cells, accounting for approximately 2.7% of all imaged cells (10 out of 370) were characterized by increased EGFR expression two- to three-fold compared to the rest of the cell population; these were termed EGFR-enriched cells. These cells mostly expressed HER2 and EGFR in clustered regions, but also on LMPs (see EGFR enriched cell in Figure 2B and Supplemental Movie S4).

**Figure 3:**
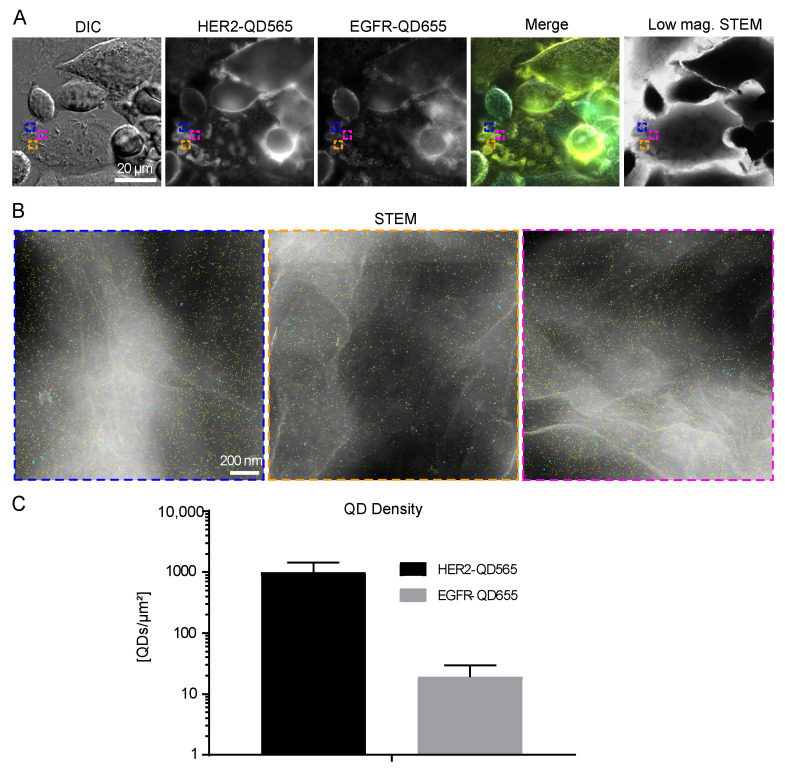
HER2 and EGFR expression on large membrane protrusions. (**A**) Correlative fluorescence and low magnification STEM of SKBR3 cells labeled for HER2-QD565 and EGFR-QD655. Light microscope images were acquired using a 63× oil objective. The same region is shown in the STEM image at M = 800×. Colored rectangles depicted in (**A**) indicate the magnified areas shown in (**B**) acquired with dark field STEM at M = 120,000×. Individual quantum dots (QDs) are visible and outlined in cyan (EGFR labeled with streptQD655) and yellow (HER2 labeled with streptQD565). (**C**) Mean QD densities for HER2 and EGFR for all analyzed large membrane protrusion regions with standard deviation indicated. Colors in the merged images: yellow for HER2 and cyan for EGFR. Scale bars: 20 µm (**A**) and 200 nm (**B**).

**Figure 4:**
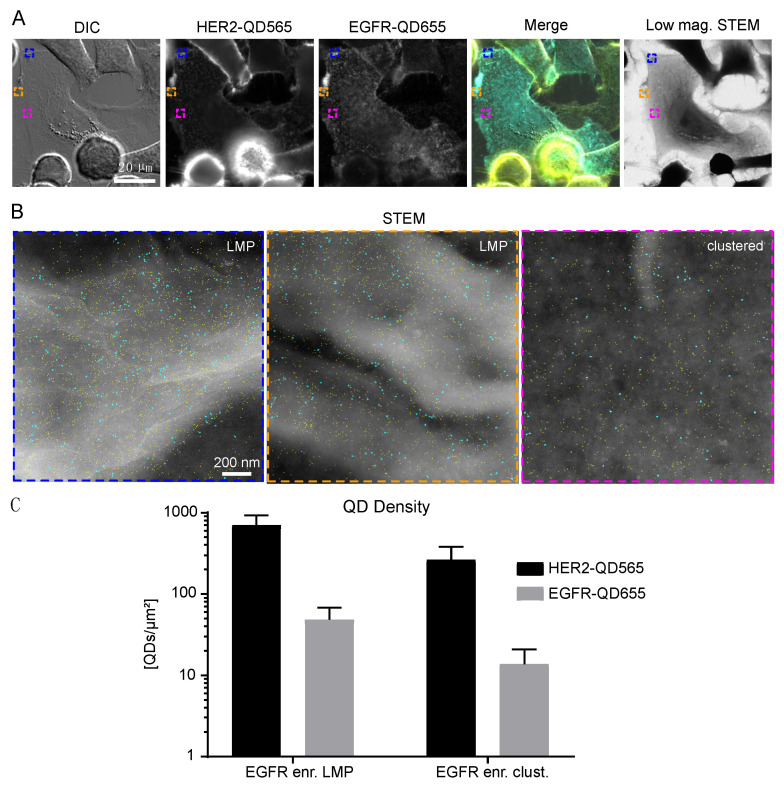
HER2 and EGFR expression on SKBR3 EGFR enriched cells. (**A**) Correlative fluorescence and low magnification electron microscopy of SKBR3 cells labeled for HER2-QD565 and EGFR-QD655. Light microscope images were taken with a 63× oil objective. The same region is shown for the low magnification brightfield STEM image, M = 800×. Colored rectangles depicted in (**A**) indicate the magnified areas shown in (**B**) acquired with annular dark field STEM at M = 120,000×. Individual QDs are visible and outlined in cyan (EGFR labeled with streptQD655) and yellow (HER2 labeled with streptQD565). (**C**) Mean QD densities for HER2 and EGFR for all analyzed EGFR enriched regions (large membrane protrusions and clustered) with standard deviation indicated. Colors in the merged image in (**A**): yellow for HER2 and cyan for EGFR. Scale bars: 20 µm (**A**) and 200 nm (**B**).

Next, the aforementioned cell subtypes were analyzed using scanning transmission electron microscopy (STEM). To this end, the microchips carrying the labeled cells were covered by multilayer graphene to prevent electron beam-induced damaging during acquisition and drying in the applied vacuum [43,44]. First, overview images were acquired with low magnification (*M =* 800) brightfield STEM, allowing the previously fluorescence imaged cells to be re-identified (Figure 2A,B). Next, dark field STEM images (*M =* 120,000) were acquired from regions of interest (LMPs, EGFR enriched, clustered and flat regions (Figure 3 and Figure 4, Supplemental Figures S8 and S9).

### 2.3. Large Membrane Protrusions Contain most HER2 Receptors

LMPs are described as membrane structures that are associated with high signaling activity, receptor trafficking, and therapy resistance [31,34,45]. Indeed, high levels of clustered HER2 were observed on these structures (Figure 3A). STEM analysis of these structures revealed the presence of individual HER2 receptors (marked yellow) on LMPs (appearing white) (Figure 3B). Amongst the HER2 receptors, both single- and dimeric EGFR receptors were detectable. QD-labeled receptor densities were found to be (9.9 ± 4.5) × 10^2^ QDs/µm^2^ for HER2 and (2 ± 1) × 10^1^ QDs/µm^2^ for EGFR on LMPs (Figure 3C and Table 1). 

### 2.4. EGFR Expression on SKBR3 is Limited to a Subgroup and to Large Membrane Protrusions

Next, the identified subgroup of EGFR enriched cells was analyzed, and it was found that EGFR and HER2 expression was mostly found in clustered regions but was also be found on LMPs (Figure 4A,B). The density of EGFR receptors was highest for this subgroup for both the LMPs and clustered regions (Table 1). The number of HER2 receptors on LMPs of EGFR-enriched cells was slightly reduced when compared to LMPs of bulk cells, whereas their expression on the clustered regions was comparable (Supplemental Figure S8 and Table 1). The lowest number of EGFR and HER2 receptors were found on bulk flat regions (Supplemental Figure S9 and Table 1).

**Figure 5:**
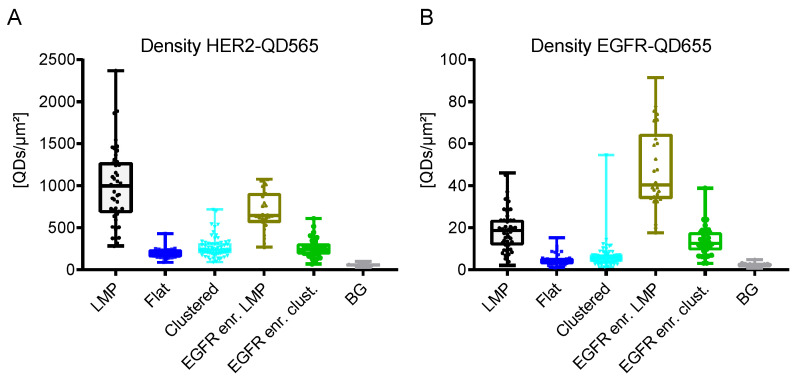
QD density and distribution analyses for SKBR3 cells dually labeled for HER2 and EGFR. (**A**) HER2-QD565 density and (**B**) EGFR-QD655 density for the different cell subtypes for SKBR3. Box-and-whisker plots indicate the mean labeled receptor densities, the lower and the upper quartiles, and the observed min to max values. Each dot represents the labeled receptor density from one image (see also Table 1). BG Background regions without cells.

Taken together, the HER2 receptor density on SKBR3 cells was determined to range from 2–10 × 10^2^ QDs/µm^2^ (Table 1 and Figure 5A). The highest density of HER2 receptors was found on LMPs (bulk cells > EGFR enriched cells), followed by clustered regions and flat regions. The density of EGFR was a factor of 20 lower ranging from 0.5–5 × 10^1^ receptors/µm^2^ (Table 1 and Figure 5B) with EGFR expression being limited to EGFR-enriched cells and LMPs. For clustered and flat regions of bulk cells, EGFR expression was almost absent.

## 3. Discussion

Aberrant growth factor receptor signaling by members of the EGFR family is linked to cancer and all four members (EGFR, HER2, HER3 and HER4) were shown to contribute to cancer cell growth, migration and metastasis formation [5]. However, the growth factor receptors do not act as single proteins but as dimers, both in homo- and heterodimeric fashion. Over the last few years, it has become increasingly clear that heterodimers play a decisive role in the development of resistance to therapy [5]. This makes it all the more important to develop new tools for single cell analysis at the protein level. As a proof of concept, we established a dual labeling protocol for HER2 and EGFR allowing parallel correlative light and electron microscopic examination of these receptors. Our previously published protocol for HER2 labeling [34] has been extended and optimized to include EGFR co-labeling with two subsequent, in-between saturated/quenched, biotin-streptavidin labeling reactions. Protocol optimization mainly involved the specific fixation conditions. Fixation of cells can be achieved by different means, with the choice of the fixation reagent depending on the research question and readout method. Here, the goal was to I) achieve preservation of membrane structures like protrusions (ruffles), and II) prevent fixation-induced labeling artifacts like label-induced receptor dimerization or non-specific binding of primary or secondary labels via the fixative [36,47,48,49], while allowing correlative fluorescence microscopy and STEM. As a standard fixative for immunolabeling, a 2–4% aqueous solution of (para)formaldehyde is generally used for 15–30 minutesat room temperature [36,48,49]. However, this fixation is insufficient for complete immobilization of all proteins in the membrane making it difficult to study membrane proteins such as receptors at the single-molecule level [49]. Glutaraldehyde is a stronger fixative than formaldehyde, and is frequently used for electron microscopy, but rarely used for immunolabeling approaches as it forms auto-fluorescent structures impairing fluorescence analysis [50], and is known to lead to deformation of alpha-helix structure in proteins [38], potentially reducing the subsequent binding of affinity ligands. Therefore, a protocol was developed consisting of a combination of both fixation reagents aiming at sufficient preservation of membrane proteins, the underlying cytoskeleton, and immobilization of HER2 receptors (stopping diffusion of the receptor in the membrane) without causing auto-fluorescence. Furthermore, our used mono-biotinylated primary labels (EGF-biotin and anti-HER2 Affibody biotin) ensured that mono label attachment to the respective receptors ensuring that the detected labels report the presence of induvial receptors. Given the availability of suited primary labels for HER3 and HER4, our protocol could be adapted or extended for the detection of these receptors as well.

The used QDs exhibit the advantage of a combination of high charge density, as needed for enhanced contrast in STEM, and a brighter emission of fluorescent light, compared to conventional fluorophores. The correlative approach allows screening and identification of cells of interest via light microcopy before starting a detailed analysis of single cells at the single protein level via electron microscopy [34]. The use of quantum dots for double or multiplex-labeling has been reported in earlier studies using light microscopy [51,52,53,54], as well as in a few studies using correlative microscopy [39,40,55], proving that this method is principally also applicable to other proteins, including intracellular proteins, after permeabilization of the plasma membrane [56].

The absolute numbers of labeled receptors ranged from 2–10 × 10^2^ QDs/µm^2^ for HER2 and 0.5–5 × 10^1^ QDs/µm^2^ for EGFR (Table 1). The amounts of detected QD labels are lower than the actual number of receptors due to non-optimal labeling efficiency. Using a similar protocol for single labeling exhibited 80% for the (smaller) QD565, respective 50% for the (larger) QD655, for HER2 [46]. It can thus be assumed that the observed label densities need to be increased by approximately these percentages to obtain the actual receptor densities. The observed receptor densities depended on the cell phenotype, on the single cell level, and on the different plasma membrane regions. Most HER2 receptors were found on LMPs, such as dorsal or lateral membrane ruffles [31,34]. These regions are known to be areas of high signaling activity expressing not only HER2 [31], but also downstream effectors like AKT [31]. In addition, LMPs are low for calcium signaling that prevents HER2 internalization [31,57,58].

While most SKBR3 membrane regions had a low EGFR density (Table 1), an enrichment for EGFR expression was seen for the LMPs (Figure 3). The formation of LMPs is indeed suggested to be initiated by the formation of EGFR/HER2 or HER2/HER3 heterodimers and this hypothesis is supported by the fact that treatment of SKBR3 cells with the EGFR and HER2 dual specific inhibitor, lapatinib, reduced the number of LMPs [31]. Our finding that EGFR expression is present on the LMPs but low in clustered regions supports this hypothesis.

Besides the LMPs, positive HER2 expression was also observed on smaller membrane deformations (see, for example, Figure 3 and Figure 4, Supplemental Figure S8). These HER2 clustered regions, have been described for HER2 overexpressing breast cancer cell lines and patient samples [37,59]. In contrast to LMPs, neither active HER2 signaling, nor the formation of EGFR-HER2 or HER2-HER3 dimers are required for the formation of these membrane deformations but are the consequence of the increased amount of HER2 receptors being present at the membrane [37]. Compared to LMPs, the observed labeled HER2 densities for the clustered regions was almost a factor of four lower ((2.7 ± 1.2) × 10^2^ QDs/µm^2^ vs. (9.9 ± 4.5) × 10^2^ QDs/µm^2^) (Figure 5A and Table 1). The clustered regions can, therefore, be considered as the default arrangement of HER2 receptors in the cell membrane being overexpressed as is the case on SKBR3 cells and its formation being independent of EGFR expression. This finding is further supported by the fact that for the clustered regions EGFR expression was very low (Table 1).

An unexpected finding was the discovered subpopulation of EGFR-enriched cells within the SKBR3 cells (Figure 4). These cells were characterized by a two to threefold enriched EGFR expression compared to the other cells, and expressed HER2 mostly in clusters and on LMPs. These cells accounted for approximately 2.7% of the cell population.

It is known that actin-rich LMPs provide resistance to endocytosis of growth factors by impaired calcium flux [31,57], and that HER2-EGFR heterodimers resist endocytosis [60]. EGFR-enriched cells might therefore offer a survival benefit during therapeutic stress, being on one side more resistant to receptor internalization and on the other side by constant generation of pro-survival signals through the accumulated EGFR-HER2 receptor clusters on LMPs.

Low abundance within the cell population, resistance to therapeutic approaches and long-term survival are characteristics of and fit the description of cancer stem cells in breast cancer [61]. Although the amount of breast cancer stem cells within SKBR3 cells might range up to five percent [62], the here described EGFR enriched cell population is not identical but might belong to the described SKBR3 stem cell population (Figure S10).

Our results present a strategy to quantitatively study localized EGFR and HER2 expression levels in the plasma membrane. The method allows determining the surface expression level of potential dimerization partners of HER2, which is shown here for EGFR. The same labeling strategy can be used to develop double labels for EGFR or HER2 with HER3 and HER4, or in fact any combination of the HER family. HER2 was reported to dimerize also with other receptors such as the insulin growth factor receptor (IGFR), and the hepatocellular growth factor MET [63,64,65]. Double labels can be developed for those candidates as well.

Lapatinib is a dual specific inhibitor of EGFR and HER2 [9]. The fact that it is superior to the first line monotherapy with trastuzumab suggests that members of the HER family may have at least partially redundant functions. This underlines the need for a dual or multipoint therapy strategy, which is also supported by recently published reports (reviewed in [3]). Triple inhibition with antibody mixtures against EGFR, HER2 and HER3 were effective in blocking the growth of cancer cells in vitro and in vivo [66]. Similar, triple inhibition of EGFR, HER2 and HER4 via the pan-HER inhibitor neratinib was shown to be superior over lapatinib in a phase III trial for brain metastatic breast cancer [67]. In fact, second line therapy approaches and ongoing trials for (neo)adjuvant and advanced metastatic breast cancer combine chemotherapy with either trastuzumab and pertuzumab or T-DM1, or trastuzumab with small compound inhibitors like lapatinib, gefitinib or neratinib [67,68,69,70,71,72]. Thus, these second line therapeutic approaches tackle heterodimer formation or aim to block receptor activation by mono-, duo- or pan-specific EGFR and or HER inhibitors. The latter findings underline the importance of I) determining the surface expression level of potential dimerization partners of HER2 and II) determining their dimerization behavior on individual cells in order to predict the response to possible treatment options and thus the success rates of therapies.

Given the availability of suitable labeling protocols, in future works the analysis method presented here can be expanded for the detection of any HER2 heterodimer. Dimerization behavior analysis on individual cells is crucial in order to predict the response to possible treatment options and thus the success rates of therapies. Thus, our established dual specific labeling protocol would provide a valuable tool to study any HER2 heterodimerization, not only for cell lines but also, as already shown for HER2 homodimerization, for patient samples [73].

## 4. Materials and Methods

### 4.1. Materials and Chemicals Were Purchased from the Following Companies

Biotin conjugated anti-HER2 Affibody (ZHER2:342)2, anti-HER2 Affibody FITC and anti-HER2 Affibody imaging agent (from Affibody AB, Bromma, Sweden). Dulbecco’s phosphate-buffered saline (DPBS) (from Lonza, Cologne, Germany). Non-essential amino acids (NEEAs) 100× solution, streptQD (Qdot^TM^565(#Q10031MP)), Qdot^TM^655 (#Q10121MP)) streptavidin conjugate, amine group ITK QD565 (Qdot^TM^565 ITK^TM^ Amino-(PEG) (#Q21531MP)), Dulbecco’s Modified Eagle Medium (DMEM) GlutaMAX (high glucose and pyruvate), fetal bovine serum (FBS), endogenous biotin blocking kit (#E21390), EGF biotin conjugate (#E3477), Illustra NAP™-5 column, succinimidyl-[(N-maleimidopropionamido)-diethyleneglycol] ester (NHS-PEG2-Maleimide SM(PEG)2), Pierce™ immobilized TCEP-disulfid reducing gel, Sartorius™ Vivaspin™ 500 Centrifugal Concentrators, MWCO 50 kDa, (from Thermo Fisher Scientific GmbH, Dreieich, Germany). Phycoerythrin-conjugated anti-CD24 antibody, clone SN3 (ab77219) and AlexaFluor^®^ 488-conjugated Anti-CD44 antibody, clone MEM85, (pre-diluted, ab187571) (from Abcam, Cambridge, UK). Streptavidin-conjugated 40 nm gold nanoparticles (AuNPs, note that transmission electron microscopy analysis revealed ~12 nm diameter AuNP, one streptavidin per AuNP) (from Kirkegaard & Perry Lab Inc, Gaithersburg, MD, USA). High pressure liquid chromatography (HPLC) grade double distilled H_2_O, acetone, ethanol, phosphate buffered saline (PBS) 1× solution pH 7.4, sodium chloride, glycine, biotin free and molecular biology grade bovine serum albumin fraction V (BSA), and sodium cacodylate trihydrate (CB) (from Carl Roth GmbH + Co. KG, Karlsruhe, Germany). Poly-L-lysine, biotin, sodium tetraborate, boric acid, sucrose, electron microscopy grade 25% glutaraldehyde solution, superfibronectin (#S5171), 2-mercaptoethanol (from Sigma-Aldrich, Munich, Germany). Electron microscopy grade formaldehyde 16% solution (from Science Services GmbH, Munich, Germany). Normal goat serum (GS) (from Rockland Immunochemicals, Gilbertsville, PA, USA). Microcon centrifugal filter unit YM-100 membrane, MWCO 100 kDa (from Millipore Merck, Darmstadt, Germany), Micro Bio-Spin P-30 Tris Chromatography Columns P30 (from Bio-Rad Laboratories GmbH, Feldkirchen, Germany), Superose-6 10/300 GL column (from GE Healthcare Life Sciences). 4-well 35 mm compartments glass bottom dishes and well plates (6, 24, 48 and 96) for tissue culture (from Greiner Bio-One GmbH, Frickenhausen, Germany). Silicon microchips with a 50 nm thick electron transparent silicon nitride (SiN) window with dimensions of 120 × 700 µm^2^ (from Norcarda, Edmonton, AB, Canada). Multi-layer graphene (polymethyl methacrylate trivial transfer graphene), (from ACS Materials, Pasadena, CA, USA). CellStripper (from Corning, New York, NY, USA)

### 4.2. Mammalian Cell Culture

The breast cancer cell line SKBR3 (HTB-30) was purchased from ATCC, Wesel, Germany. Cells were cultured in growth medium (DMEM-GlutaMAX supplemented with 10% FBS and 1% NEAAs) and kept at 37 °C in a CO_2_ incubator, in a 5% CO_2_ water saturated, air atmosphere. Cells were passaged twice a week and not used longer than passage 25. Cells were tested for mycoplasma contamination and authenticated by single nucleotide polymorphism analysis (Mulitplexion, Friedrichshafen, Germany).

### 4.3. Coating of Microchips and Cell Culture Microscope Dishes

Microchips were prepared for cell seeding as follows: Chips were carefully removed from the gel pack, washed for two minutes in HPLC-grade acetone, five minutes in HPLC-grade ethanol, five minutes in HPLC-grade H_2_O, rinsed in HPLC-grade ethanol, then air dried under the fume hood. The following steps were also applied to cell culture microscope dishes. A plasma cleaning step with argon and oxygen was applied for 5 min to render the surfaces of microchips and dishes hydrophilic. Next, a 0.01% aqueous poly-L-lysine solution was incubated at room temperature for five minutes. After two rinses with water, the surfaces were incubated with 5 µg/mL superfibronectin diluted in PBS for two hours at 37 °C. Surfaces of chips or dishes were rinsed twice with PBS, microchips were immersed in a well of 100 µL serum-free DMEM in a 96-well plate or dishes were covered with serum-free DMEM (500 µL) until cell seeding.

### 4.4. Cell Seeding on Microchips or Cell Culture Microscope Dishes

Detailed visual protocols presenting the following steps (Section 4.4, Section 4.5, Section 4.6, Section 4.7 and Section 4.8) have been published to which the reader is referred for additional information [43,74]. SKBR3 cells were non-enzymatically detached with cell stripper for 10–15 min at 37 °C, re-suspended in growth medium, then counted and diluted to 1 × 10^5^ cells/mL. For microchips a total volume of 100 µL, corresponding to 1 × 10^4^ cells, was added to each well in a 96-well plate containing a microchip. For dish experiments, 1 mL (1 × 10^5^ cells) was added to each well of a compartment dish. Cells were allowed to attach for five minutes after which cell density was checked. In case cell density on the silicon nitride window of the chips was too low, more cells were added (2 to 5 × 10^4^ cells). After two hours, microchips were transferred to new wells pre-filled with growth medium; in the case of dishes, medium was replaced with fresh growth medium. Cells were allowed to grow for 48 h before being starved in serum-free DMEM overnight to enhance growth receptor expression on the cell surface.

### 4.5. Dual Labeling of EGFR and HER2, CD44 Immunofluorence Labeling

Fixation and labeling for cells grown on microchips were performed in a 48-well plate format with 300 µL liquid volumes for fixation or a 96-well format with 200 µL volumes for labeling. The individual steps were performed subsequently by transferring the microchips from well to well containing the mentioned solutions. For four well compartment dishes, 400 µL was used for the washing and rinsing steps, and 250 µL was used for labeling reagents. For dishes, reagents and solutions were applied by subsequent pipetting steps, all performed at room temperature.

For fixation, cells were rinsed once with serum-free medium and once with CB (0.1 M cacodylate buffer, 0.1 M sucrose pH 7.4). Cells were fixed by two-step fixation, first with 3% formaldehyde (diluted in CB) for five minutes, then with 3% formaldehyde and 0.2% glutaraldehyde (diluted in CB) for five minutes (note: glutaraldehyde was added to preserve the cellular membrane structures and to prevent ligand induced dimerization that occurs to certain extent with formaldehyde fixation only). After fixation, cells were rinsed once with CB and thrice with PBS. Non-saturated aldehyde groups were quenched with 1 M glycine-PBS solution for 10 min. After one additional rinsing step with PBS, cells were labeled. Labeling steps were incubated on a horizontal rocker with 60 rounds per minute.

EGFR receptors were labeled first, by incubation with 400 nM EGF-biotin diluted in PBS/BSA 1% for ten minutes. Unbound EGF-biotin was removed by rinsing twice with PBS/BSA 1% and washing once with PBS/BSA 1% for five minutes. Next, cells were incubated with 20 nM streptavidin conjugated quantum dots (streptQD)655 for 12 min. Non-bound streptQD655 reagent was removed with PBS/BSA 1% by rinsing twice and washing once for five minutes.

To saturate any remaining streptavidin binding site, cells were incubated with streptavidin solution (endogenous biotin blocking kit, Thermo Fisher) for five minutes. After three rinsing steps with PBS, free biotin binding sites were saturated by incubation with 30 µg/mL biotin solution (diluted in PBS/BSA 1%) for five minutes. Unbound biotin reagent was removed by rinsing thrice with PBS.

HER2 was labeled next. Cells were blocked with 1 M glycine-PBS/BSA 1%/GS 1% for 10 min followed by incubation with 200 nM anti-HER2-Affibody biotin (diluted in blocking solution) for 10 min. Unbound Affibody was removed by rinsing twice and washing once for five minutes with PBS/BSA 1%. Finally, streptQD565 were attached to the HER2-anti-HER2Affibody-biotin complexes by incubation of 20 nM streptQD565 (diluted in PBS/BSA 1%) for 12 min. Excess streptQD565 were removed by rinsing twice and washing once for five minutes with PBS/BSA 1%. After labeling, cells were immediately imaged with differential interference contrast and fluorescence microscopy.

Alternatively, cells were labeled with 200 nM anti HER2 Affibody FITC. Living cells were rinsed once with serum free medium, blocked with 1 M glycine-PBS/BSA 1%/GS 1% for 10 min followed by incubation with 200 nM anti-HER2-Affibody FITC (diluted in blocking solution) for ten minutes at 37 °C. Unbound Affibody was removed by rinsing twice and washing once for five minutes with PBS/BSA1 % at 37 °C. Cells were covered with pre-warmed CO^2^-independent medium (Thermo Fisher Scientific GmbH) and imaged directly at 37 °C. In case fixed cells were labeled with anti-HER2 Affibody FITC, the above described procedure was applied at room temperature.

After HER2, respect. EGFR labeling with QDs, the CD44 receptor was immunolabeled with Anti-CD44 and Anti-CD24 antibodies directly conjugated to fluorophores (Anti-CD24-PE and Anti-CD44-Alexa 488) diluted 1:100 in PBS/BSA1 %. Subsequently, the cells were washed three times with PBS before cells were immediately imaged with differential interference contrast and fluorescence microscopy.

### 4.6. Differential Interference Contrast and Fluorescence Microscopy

Cells grown on microchips or 4-well compartment dishes were imaged with a DMi6000B inverse microscope (Leica, Wetzlar, Germany) using the LAS FX software (version 3.6.0.20104) and a connected DFC365FX-camera. Cells grown on microchips were imaged upside down in glass bottom dish in 1 mL PBS/BSA 1%. Images were acquired with a 1392 × 1040 resolution with a 20× (HC PL FLUOTAR L 20×/0.40 DRY) or a 63× (HC PL APO 63×/1.40 OIL) or 100× (HC PL APO 100×/1.40 Oil CS2) objective. The following channels were acquired: differential interference contrast (DIC), fluorescence optimized for streptQD565 (filter QDot565, size K ex. 425/50 nm; dichroic 505 nm; em. 565/30 nm), fluorescence optimized for streptQD655 (filter QDot655, size K ex. 425/50 nm; dichroic 510 nm; em. 655/30 nm) and for GFP (filter L5 ex. 460–500 nm/em. 512–542 nm). Tiled images and Z-stack images were automatically recorded, exported as 16 bit TIFF grayscale format, analyzed and processed with Fiji ImageJ (version 1.52p, National Institutes of Health, Bethesda, MA, USA). After imaging, microchips were post-fixated by incubation in 2% glutaraldehyde for 10 min followed by one rinse with CB and three rinses with PBS. Microchips were stored in PBS/BSA 1%/NaN_3_ 0.02% at 4 °C until electron microscopy analysis.

### 4.7. Graphene Enclosure of Microchips for Electron Microscopy

To maintain a hydrated state and protect from electron beam-induced damage, the microchip-grown, labeled cells were covered by a three to five-layer sheet of graphene. The preparation procedure and transfer of graphene to NaCl crystals and the microchip preparation has been previously described in detail [43]. Briefly, a small piece of NaCl carrying enough graphene to cover the electron transparent window of the microchip was dissolved in water, leaving the graphene floating on the surface of the water. The graphene was captured with a metal loop floating on a small water droplet. The microchip to be covered was rinsed thrice with water to remove any residual PBS/BSA 1%/NaN_3_ 0.02% and attached to the lower surface of the metal loop with graphene water droplet. Excess water between the graphene layer and the microchip was carefully removed with filter paper under a binocular microscope.

### 4.8. Scanning Transmission Electron Microscopy (STEM)

Graphene-covered microchips were mounted in a standard electron microscope holder (JEOL) and imaged with a scanning transmission electron microscopy (STEM, ARM 200F, JEOL, Akishima, Japan) at 200 keV electron beam energy. The image resolution was 2048 × 2048 pixels with overview images acquired in low magnification brightfield mode at *M* = 800× (pixel size of 0.13 × 0.13 µm), and annular dark field STEM images acquired at *M* = 120,000× (pixel size of 0.83 × 0.83 nm) using image acquisition software (Digital Micrograph, Version 3.30.2016.0, Gatan, CA, USA). A condenser lens aperture of 20 µm was used, and a probe size of 2C corresponding to a probe current of 175 pA, and pixel dwell time of 18 µs. The maximal electron dose amounted to 280 e^−^Å^−2^ within the limit of radiation damage for these samples [43]. The electron beam was set to a beam convergence angle of 13.2 mrad and the STEM detector opening angle β_in_ − β_out_ = 68–280 mrad.

### 4.9. Particle Analysis

Images recorded in high magnification STEM mode were analyzed with image processing scripts (Fiji ImageJ, version 1.52p) [34]. The script functioned as follows. First, the image was subjected to a Gaussian filter with a radius of 1.5 pixels to reduce noise. Second, a Fourier filter for negation of image background was applied, then the image was binarized with an automatic threshold with maximal entropy setting. To detect different sized QDs, the minimal and maximal pixel size for the large QDs (QD655) was set manually by the user (according to a QD size of 11 nm ± 2 nm) and automatically adopted for the small QDs (QD565) by the script applying the “detect particle” tool of ImageJ. Output files contained x, y coordinates for each detected large and small QDs as separate lists. These files were further used to calculate the QD densities of each image. The source of the scripts is available on request.

### 4.10. Statistical Analysis

Column statistics on particle densities, their calculation, and plotting of data was done with scientific plotting software (GraphPadPrism 7.0, GraphPad, SanDiego, CA, USA). Data is presented as bar graphs indicating the mean, with standard error of the mean as box whisker plots indicating the mean receptor densities with mix to max projections.

## Figures and Tables

**Table 1:** STEM statistics for dually labeled and analyzed SKBR3 cells and images. For each cell type (bulk or EGFR enriched) and region (LMP, clustered or flat), the number of analyzed cells, images, particles (QD565 and QD655) and the calculated densities for QD-labeled HER2 and EGFR is given. n.a. not applicable. See Supplemental Table S1 and [46] for errors of QD detection.

Cell Subtype	Cells	Images	Imaged Area [µm^2^]	Particles QD565	HER2-QD565density/µm^2^ (Mean ± STD) × 10^2^ (QDs/µm^2^)	Particles QD655	EGFR-QD655density/µm^2^ Mean ± STD × 10^1^ (QDs/µm^2^)
Bulk, clustered regions	31	68	196	52,744	2.7 ± 1.2	1301	0.6 ± 0.6
Bulk, large membrane protrusions	21	45	128	128,847	9.9 ± 4.5	2478	2 ± 1
Bulk, flat regions	15	31	89	18,212	2.0 ± 0.7	448	0.5 ± 0.3
EGFR enriched, large membrane protrusions	10	26	72	52,675	7.0 ± 2.3	3606	5 ± 2
EGFR enriched, clustered regions	10	34	98	26,047	2.7 ± 1.2	1342	1.3 ± 0.7
Background (substrate)	n.a.	15	43	2531	0.6 ± 0.2	102	0.2 ± 0.1

## Supplementary Materials

Supplementary Materials can be found at https://www.mdpi.com/1422-0067/21/23/9008/s1.

## Abbreviations

BSA	bovine serum albumin
CB	sodium cacodylate trihydrate buffer
DIC	differential interference contrast
DMEM	Dulbecco’s Modified Eagle Medium
DPBS	Dulbecco’s PBS
EGF	epidermal growth factor
EGFR	EGF receptor
EGFR-QD655	EGFR coupled to EGF conjugated biotin coupled to streptavidin QD655
FBS	fetal bovine serum
FITC	Fluorescein isothiocyanate
GA	glutaraldehyde
GS	goat serum
HER2	human epidermal growth factor receptor 2
HER2-QD565	HER2 bound to biotinylated anti-HER2 Affibody coupled to streptavidin QD565
HPLC	high pressure liquid chromatography
LMP	large membrane protrusion
NEAAs	non-essential amino acids
PBS	phosphate buffered saline
PI3K	phosphoinositide 3-kinase
PLL	poly-L-lysine
QD	quantum dot
SF-DMEM	serum-free DMEM
STEM	scanning transmission electron microscopy
streptQD	streptavidin quantum dots
