# Supplementary material for: EGFR Expression in HER2-Driven Breast Cancer Cells
Source: Int J Mol Sci. 2020 Nov 27;21(23):9008. doi: 10.3390/ijms21239008 (PMC7729501; doi:10.3390/ijms21239008)
Supplement: Supplementary file 1 [file ijms-21-09008-s001.zip › ijms-1008180-supplementary update/ijms-1008180-Supplementary Imformation.pdf]

International Journal of
Molecular Sciences

MDPI

Supplementary Information

EGFR Expression in HER2-Driven Breast Cancer Cells

Florian Weinberg, Diana B. Peckys and Niels de Jonge

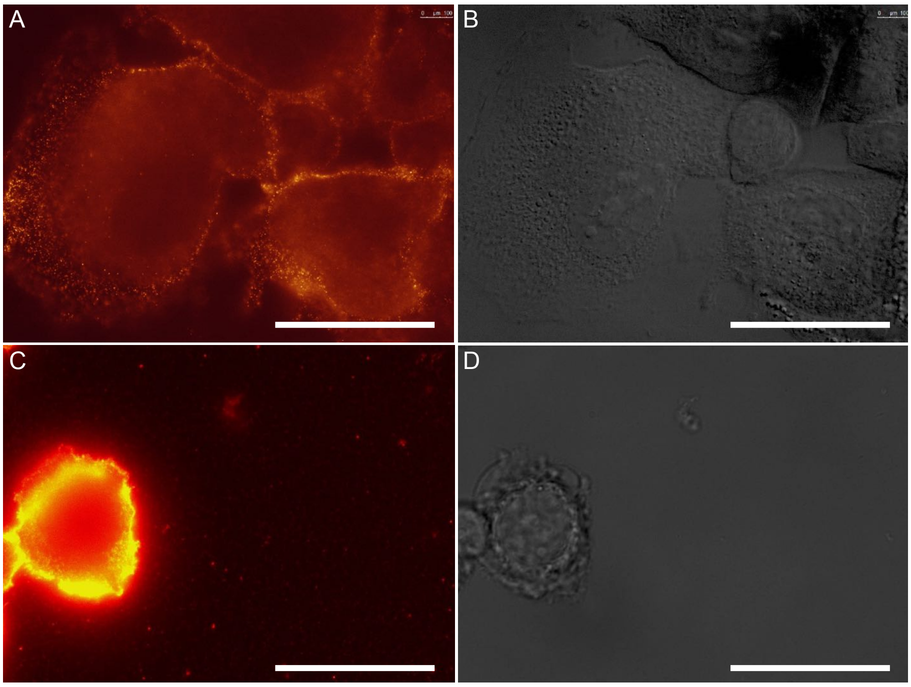

Figure S1: Epidermal growth factor receptor (EGFR) type 2 (HER2) labeling performed with a 1-step Affibody-QD565 protocol yielded a lower labeling efficiency when compared to the 2-step protocol. (A) Fluorescence microscopy, and (B) corresponding differential interference contrast (DIC) image of SKBR3 cells showing specific labeling of HER2 after incubating live cells with ~1.2 μM anti-HER2 Affibody biotin conjugated to streptavidin-coated quantum dot (QD) type 565 nm (Aff-QD565), based on the anti-HER2-Affibody imaging agent (HER2-Aff-IA), for 30 minutesat room temperature (RT). (C) Significantly higher fluorescence signals are visible on SKBR3 cells processed with the sequential 2-step protocol, as performed for the double labeling experiments presented in the main manuscript. (D) The corresponding DIC image of (C). The lower labeling efficiency in (A) was likely due to a higher steric hindrance of the Aff-conjugated QD probe, compared to the streptavidin QD (streptQD) compound used in the 2-step protocol. The Aff-QD label was made by activating 1.6 μM amine group ITK QD565 with 1 mM succinimidyl-[(N-maleimidopropionamido)-diethyleneglycol] ester (NHS-PEG2-Maleimide SM(PEG)2), and in parallel, reducing 150 μM HER2-Aff-IA with immobilized Tris [2 -carboxyethyl] phosphine hydrochloride (TCEP) Disulfide Reducing Gel, both reactions were performed at RT, and lasted 2 h. The activated amine-QDs were washed through an Illustra NAP™-5 column and incubated together with the reduced HER2-Aff-IA, for 2 h, at RT. The conjugation reaction was quenched by adding 10 mM 2-mercaptoethanol and the Aff-QD solution was concentrated with Vivaspin 500 devices (50 kDa MWCO). Purification of the Aff-QQ solution was performed with aqueous size exclusion chromatography using high performance liquid chromatography (HPLC) on a Superose-6 10/300 GL column (GE Healthcare Life Sciences), using phosphate buffered solution (PBS), pH 7.4 as the mobile phase and a flow rate of 0.5 mL/minute LM imaging was done for (A) and (B) with a 100× -, and for (C) and (D) with a 63× oil immersion objective. Scale bars: 50 μm.

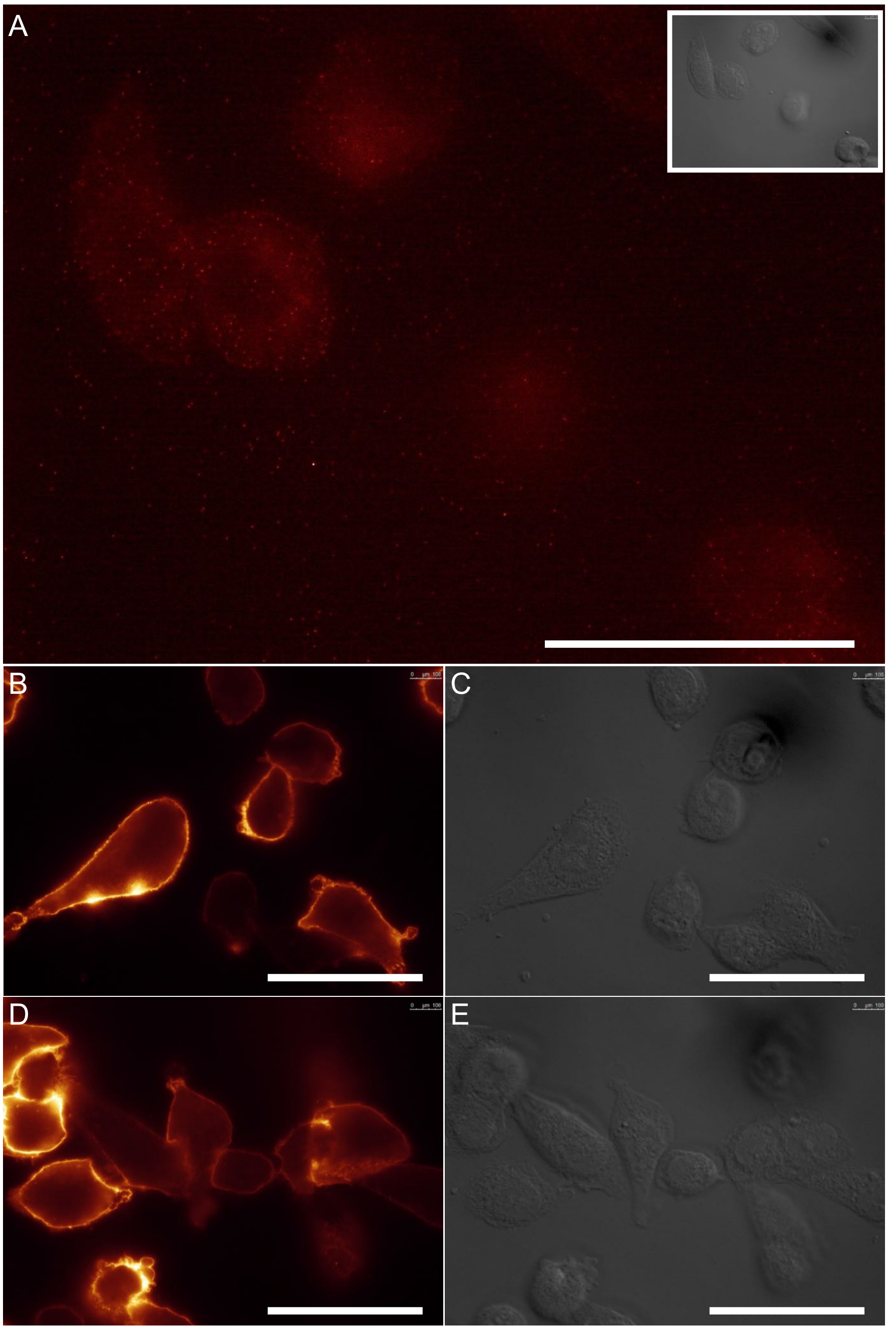

Figure S2: Comparison of two different EGFR 1-step labeling protocols with the EGFR 2-step protocol, performed in highly EGFR-overexpressing MDA-MB-468 cells, after fixation with 3% FA. (A) Fluorescence image of the cells after 30 minutes of incubation, at RT, with a 1-step Aff-QD565 probe, showing a very low labeling efficiency for EGFR, i.e., only few single QD fluorescence signals were discerned. EGFR was labeled with ~1.2 μM Aff-QD565, made with a similar protocol as the 1-step HER2-QD probe shown in Figure S1, but with EGFR-Aff-IA instead of HER2-Aff-IA. The density of QDs on the cells is only 2–4-fold higher than the low non-specific QD density on the underlying substrate. In addition to a probably higher steric hindrance of the Aff-QD565 probe compared to the streptQD probe, it also seemed that the Anti-EGFR Affibody was not suited to bind to EGFR on fixed cell, as would be necessary for EGFR-HER2 double labeling experiments. The insert shows the corresponding DIC image. (B) In contrast to the Aff-QD565 probe used in (A), here, the EGFR labeling was done with a 1-step protocol using an EGF-QD565 probe, made with a protocol published earlier [1] yielding a much higher labeling efficiency. (C) The corresponding DIC image of (B). (D) A similarly high labeling efficiency for EGFR was achieved by applying the 2-step protocol with EGF-biotin, followed by incubation with streptQD565, as performed for the double labeling experiments presented in the main manuscript. (E) The corresponding DIC image of (F). Light microscopy was done with a 100× oil immersion objective. Scale bars: 50 μm.

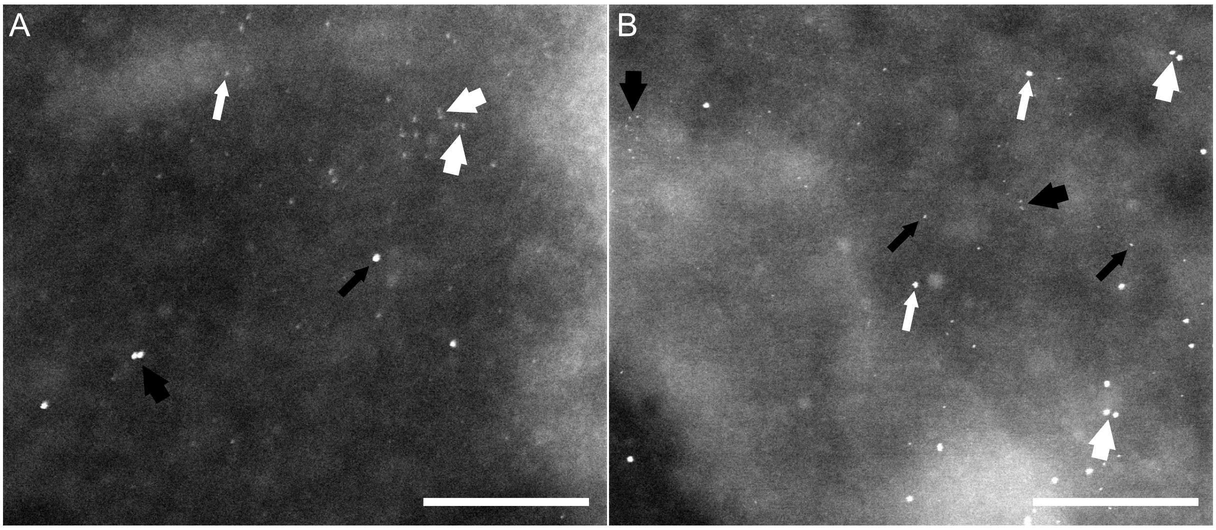

Figure S3: Exemplary results from double labeling experiments of EGFR and HER2 using gold nanoparticle (AuNP) probes in combination with QD probes on SKBR3 cells. Shown are liquid phase dark field electron microscopy (STEM) images (magnification, M = 75,000×) recorded in an environmental scanning electron microscope (ESEM) with a scanning transmission electron microscopy (STEM) detector (details of the imaging procedure are described [2]). Black arrows point to labeled EGFR, white arrows point to HER2, whereby receptor homodimers are indicated by thick arrows, and thin arrows point to single labeled receptors indicating true monomers or single-labeled dimers. A 1-step protocol was first applied to label HER2, followed by labeling EGFR with another 1-step protocol. (A) Live cells were first incubated at RT with 5 nM HER2-QD655, then with 5 nM EGF-AuNP; each step lasted 5 min as expected, due to the much higher ratio of expressed HER2 versus EGFR in SKBR3 cells, the STEM image revealed more QDs than AuNPs, thus more labeled HER2 than EGFR. In addition, homodimers of both receptors were present. The HER2-QD655 probe was made by incubating biotinylated Anti-HER2 Affibody with StreptQD655 in a 20:1 ratio, at 35 °C, for 1 h, followed by centrifugation through size exclusion spin columns (Microcon YM 100). The EGF-AuNP probe was made as described earlier [2]. (B) Similar double labeling as in (A), but with switched AuNP in the probes. Thus, the first labeling was performed with 5 nM HER2-AuNP for 15 min at RT, followed by incubation with 5 nM EGF-QD655 for 5 min Compared to (A), a higher density of bound AuNP was visible, which can be expected as this AuNP labels now HER2. Notably, switching the AuNP revealed that the density of labeled EGFR was higher, compared to the density in (A), thus pointing to the significantly higher labeling efficiency of QD probes versus AuNP probes. The HER2-AuNP probe was made by incubating biotinylated Anti-HER2 Affibody with Streptavidin-conjugated AuNP (~12 nm diameter, from Kirkegaard & Perry Lab Inc, Gaithersburg, MD, USA) in a 10:1 ratio at 35 °C for 1 h, followed by centrifugation through size exclusion spin columns (Micro Bio-Spin P-30 Tris Chromatography Columns P30) (4 repeats). Scale bars: 500 nm.

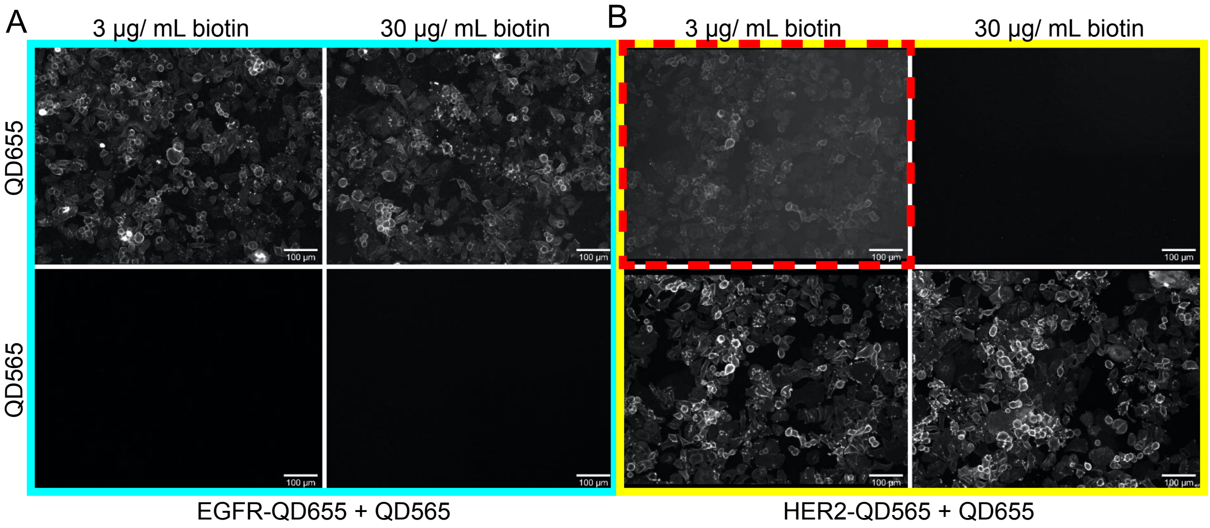

Figure S4: Biotin blocking concentration test and non-specific binding test for QDs. SKBR3 cells were grown on glass bottom dishes, allowed to grow for 48 h, fixed, and labeled for EGFR-QD655 (A, blue outline) or HER2-QD565 (B, yellow outline). Cell surfaces were blocked with either 3 µg/mL or 30 µg/mL biotin solution before QD565 (A) or QD655 (B) were incubated and their non-specific attachment to the first label was examined by fluorescence microscopy. Note that for 3 µg/mL biotin blocking, an insufficient blocking was observed for the QD655 (B upper left) reported by the non-specific attachment to the HER2-QD565 label (dashed red outline). Vice-versa 3 µg/mL biotin blocking was sufficient to prevent non-specific attachment of QD565 to the EGFR-QD655 label (A lower left). With 30 µg/mL biotin, no cross-binding of either QD was observed. Neither 3 µg/mL nor 30 µg/mL biotin incubation impaired fluorescence of primary label. Images were acquired with a 20× objective. Scale bars: 100 µm,

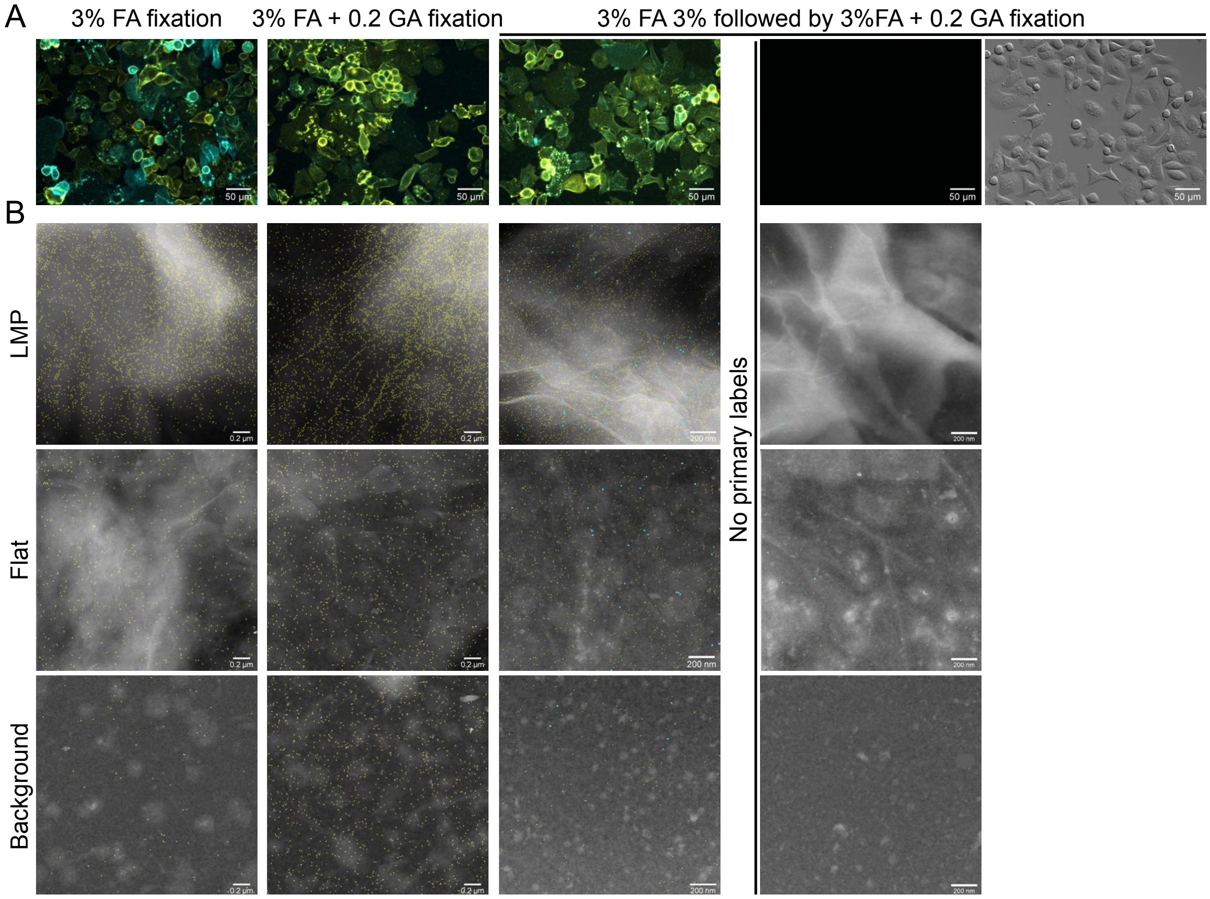

Figure S5: Example fluorescence and STEM images of differently fixed SKBR3 cells. Cells were either fixed with 3% formaldehyde for 10 min or with 3% formaldehyde and 0.2% glutaraldehyde for 10 min or with 3% formaldehyde for 5 min and subsequently with 3% formaldehyde and 0.2% glutaraldehyde for 5 min. (A) SKBR3 cells grown on a glass dish were fixed as indicated and dually labeled with HER2-QD565 and EGFR-QD655. For the cells on the very right, primary labels were omitted. Images were acquired with a 20× objective and overlays for the fluorescent channels depicting HER2-QD565 colored in yellow and EGFR-QD655 colored in cyan. (B) Representative images acquired in STEM mode with automatically detected QDs in yellow for large membrane protrusions or flat regions on the cells or background images from regions outside of cells. For cells fixed with 3% formaldehyde for 10 min or with 3% formaldehyde and 0.2% glutaraldehyde for 10 min only HER2 was labeled with streptQD655, for cells fixed with 3% formaldehyde for 5 min and subsequently with 3% formaldehyde and 0.2% glutaraldehyde HER2 was labeled with streptQD565 and EGFR was labeled with streptQD655. Individual QDs are visible and outlined in cyan (EGFR coupled streptQD655) and yellow (HER2 coupled streptQD565). Scale bars in (A): 50 µm, in (B): 0.2 µm.

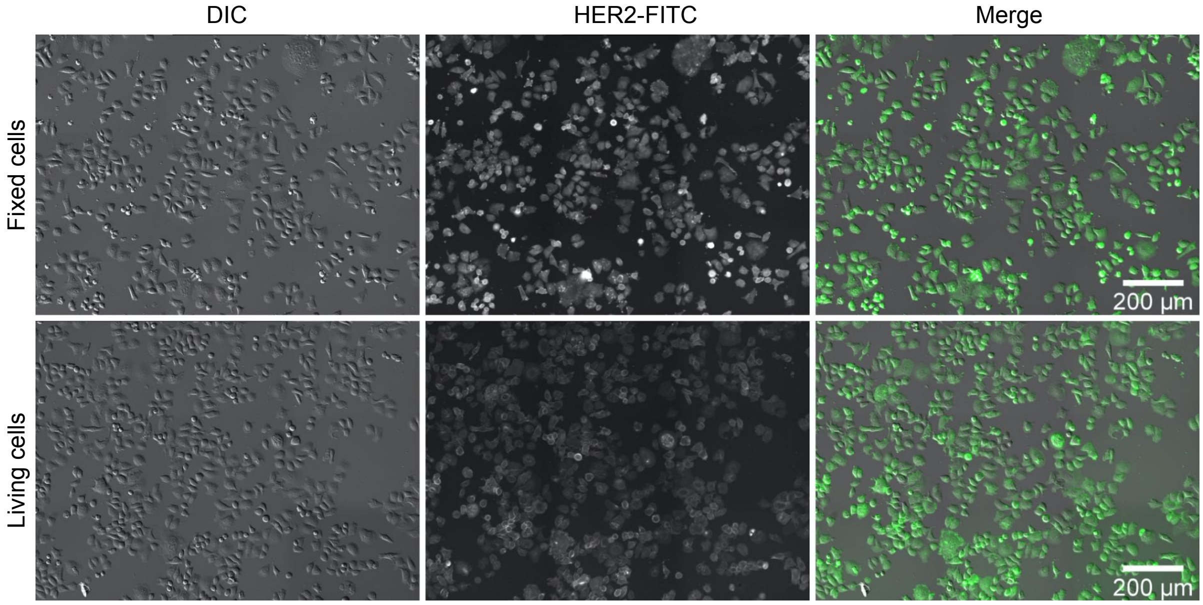

Figure S6: Fixation did not alter the anti-HER2-Affibody binding capacity. SKBR3 were seeded on glass bottom dishes and allowed to grow for 24 h. Cells were then either fixed (upper panel) and labeled with anti-HER2-Affibody-FITC (Fluorescein isothiocyanate) or directly labeled with the anti-HER2-Affibody-FITC. Cells were imaged in CO_2 independent medium at 37 °C with a 20× objective. Image settings were kept the same. No impairment for HER2 labeling was observed for fixed cells. Scale bars: 200 µm.

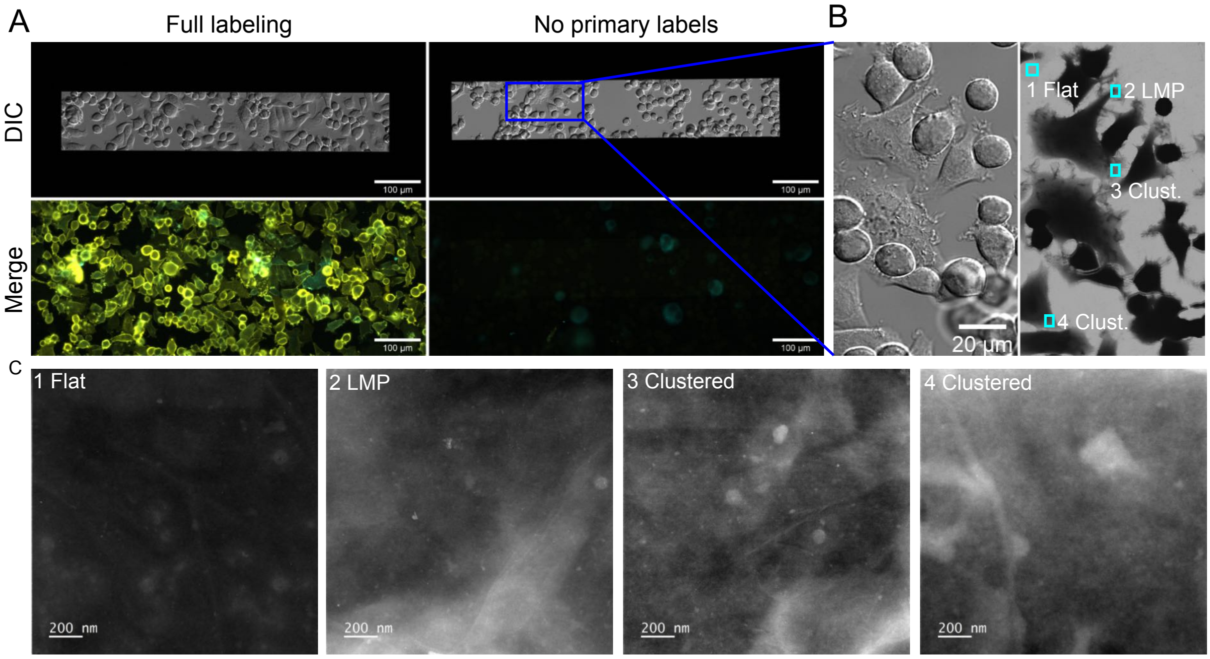

Figure S7: Off-target binding/non-specific binding of QD565 and QD655 to fixed cells without primary label. (A) SKBR3 cells were seeded on microchips and allowed to grow for 48 h. After fixation, the dual labeling protocol was applied. Note: for the left, the same microchip as the one in Figure 2 is shown. For the microchip on the right, only streptavidin QDs were incubated, no primary labels (EGF biotin and anti-HER2 Affibody biotin). For QD565, no non-specific binding to the cell surface was observed and to a little extent also to the cell surface for QD655. Images were acquired with a 20× objective, and stitched together automatically with ImageJ. Imaging settings were identical. Brightness and contrast settings were identical for all channels, and were increased to the same extent for the merged images. Colors in merged images are yellow for HER2-QD565 and cyan for EGFR-QD655. (B) Zoomed-in region outlined in blue in (A) acquired with a 63× objective for light microscopy (left) and the corresponding region acquired in low magnification bright field STEM mode with M = 800× (on the right). (C) STEM images

acquired with M = 120,000× of the indicated regions in B). No streptQDs were detectable for different regions. Scale bars: 100 μm (A), 20 μm (B), 200 nm (C).

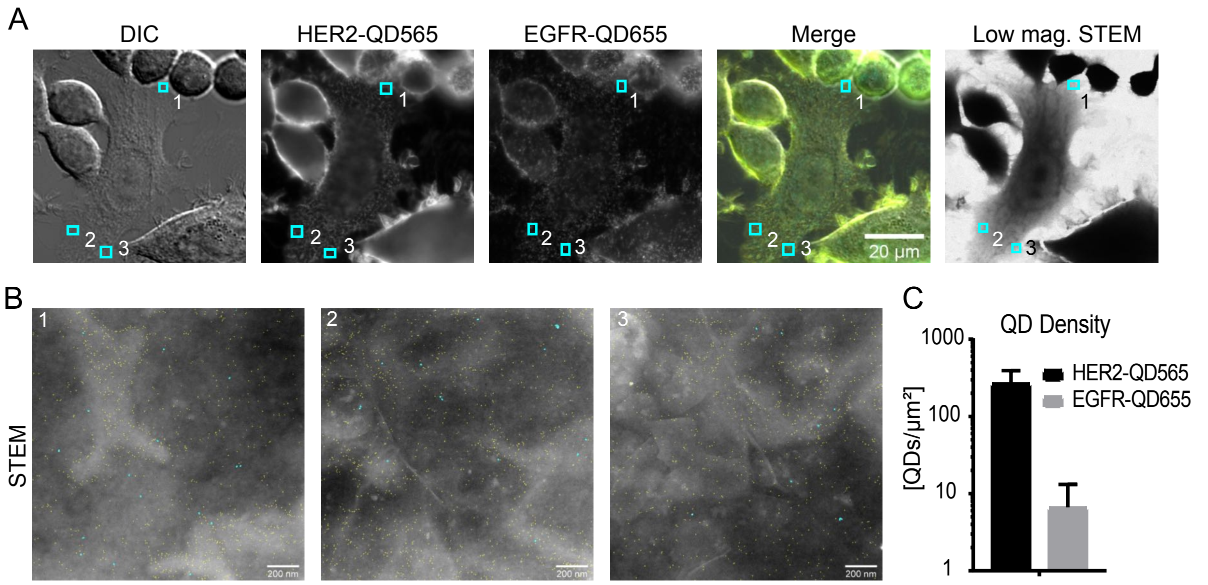

Figure S8: HER2 and EGFR expression on SKBR3 clustered regions. (A) Correlative fluorescence and low magnification STEM of SKBR3 cells labeled for HER2-QD565 and EGFR-QD655. Light microscope images were acquired with a 63× oil objective. The same region is shown for the low magnification brightfield STEM image, M = 800×. Rectangles depicted in (A) (1–3) indicate the magnified areas shown in (B) acquired with dark field STEM at M = 120,000×. Individual QDs are visible and outlined in cyan (EGFR labeled with streptQD655) and yellow (HER2 labeled with streptQD565). (C) Mean QD densities for HER2 and EGFR for all analyzed bulk clustered regions with indicated standard deviation. Colors in the merged image: yellow for HER2 and cyan for EGFR. Scale bars: 20 μm (A) and 200 nm (B).

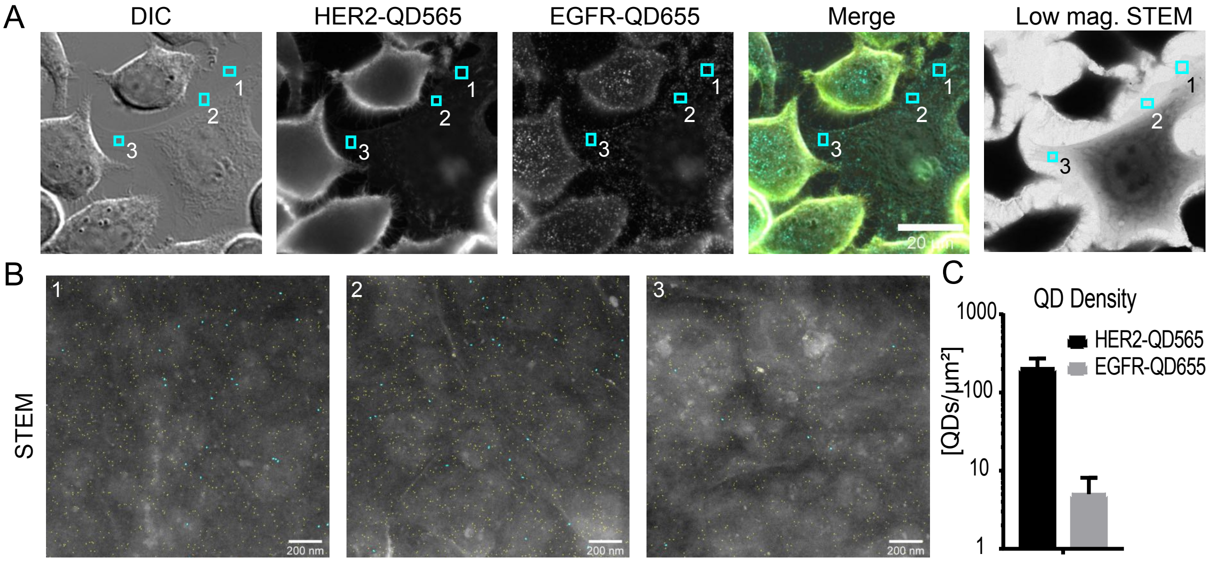

Figure S9: HER2 and EGFR expression on SKBR3 flat regions. (A) Correlative fluorescence and low magnification STEM of SKBR3 cells labeled for HER2-QD565 and EGFR-QD655. Light microscope images were acquired using a 63× oil objective. The same region is shown for the low magnification STEM image at M = 800×. Rectangles depicted in (A) (1–3) indicate the magnified areas shown in (B) acquired with STEM, M = 120,000×. Individual QDs are visible and outlined in cyan (EGFR labeled with streptQD655), and yellow (HER2 labeled with streptQD565). (C) Mean QD densities for HER2 and EGFR for all analyzed flat regions with indicated standard deviations. Colors in the merged image: yellow for HER2 and cyan for EGFR. Scale bars: 20 μm (A) and 200 nm (B).

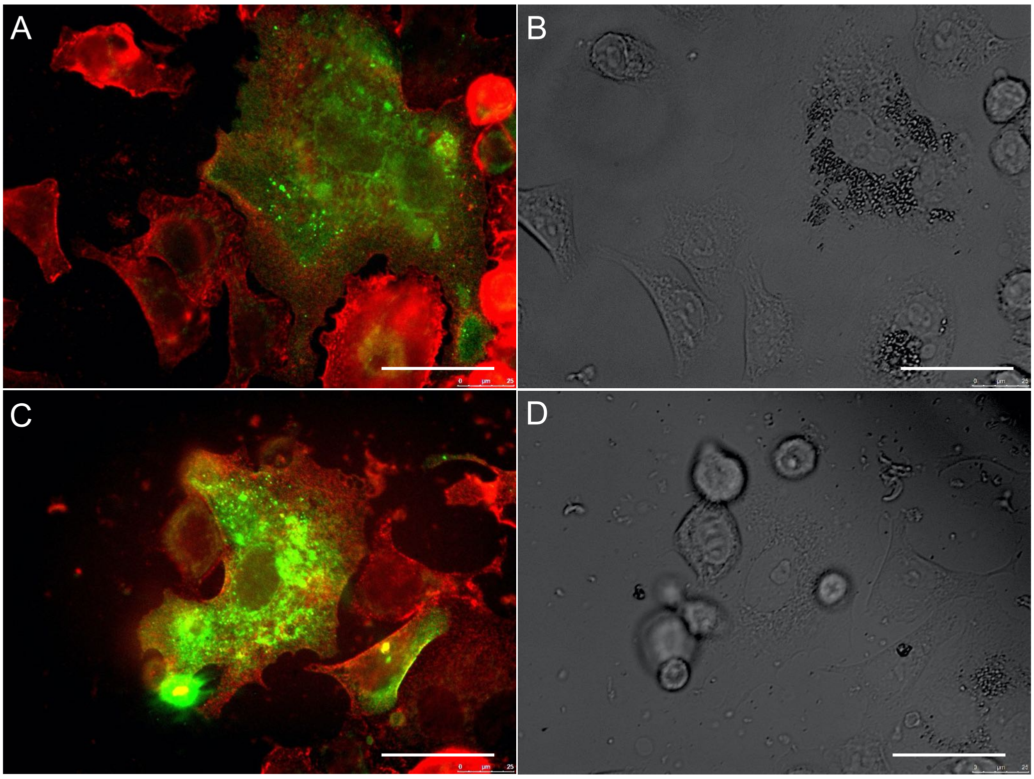

Figure S10: Double labeling of SKBR3 cells for HER2 or EGFR in combination with the breast cancer stem cell marker CD44, showing that CD44 positive cells expressed both receptors. (A) Exemplary overlay fluorescence image of SKBR3 cells with QD-labeled HER2 (red), using a 2-step labeling protocol similar to the protocol applied for the double labeling experiments in the main text, but with 5 nM streptQD655 (as in [3]), followed by incubation with a 1:100 dilution of an Anti-CD44 antibody conjugated to Alexa488 (Abcam) (green). The SKBR3 cell line was reported earlier to include ~6% of CD44+/CD24- cells [4]). Within this subpopulation of breast cancer stem cells, HER2 expression was found at varying levels. (B) Corresponding DIC image for (A). (C) Overlay fluorescence image of SKBR3 cells processed with the same CD44 labeling, but after prior 2-step EGFR-labeling, using EGF-biotin and streptQD655 (5 nM). Also in this experiment, the CD44+/CD24- subpopulation showed a large range of different EGFR expression levels. D) Corresponding DIC image for C). LM was done with a 63× oil immersion objective. Scale bars: 50 μm.

Table S1: Evaluation of the precision of automated QD detection in STEM images. The number of automatically detected QDs by the imageJ script was manually checked for detection errors. The total amount of automatically detected QDs, the number of false positive detected QDs, the number of missed QDs (false negative) and the respective error rates for the two different labels are given.

	EGFR-QD655					HER2-QD565				
#images	detected QDs	false pos.	False pos. rate [%]	false neg	False pos. rate [%]	detected QDs	false pos.	False pos. rate [%]	false neg.	False neg. rate [%]
7	456	10	2.2	14	3.1	18510	401	2.2	611	3.3

Movie S1: Z-Stack acquisition of SKBR3 cells with large membrane protrusions or clustered regions. Eight image Z-Stack (step size 2.063 µm) of dual labeled SKBR3 cells grown on a microchip. From left to right: HER2-QD565, EGFR-QD655, DIC, Merge (yellow HER2-QD565, cyan EGFR-QD655). Scale bar 20 µm. C = Cells with clustered HER2 expression pattern, LMP = cells with large membrane protrusions.

Movie S2: Z-Stack acquisition of SKBR3 cells with flat regions. Eight image Z-Stack (step size 2.063 µm) of dual labeled SKBR3 cells grown on a microchip. From left to right: HER2-QD565, EGFR-QD655, DIC, Merge (yellow HER2-QD565, cyan EGFR-QD655). Scale bar 20 µm. Arrows point to flat regions. Note that brightness and contrast have been increased to visualize the HER2 expression on the flat regions thus overexposing the fluorescence of the surrounding cells.

Movie S3: Z-Stack acquisition of SKBR3 cells with flat appearance. Eight image Z-Stack (step size 1.916 µm) of HER2 labeled SKBR3 cells grown on a microchip. From left to right: DIC, HER2-QD565, Merge (yellow HER2-QD565). Scale bar 20 µm. LMP cell with large membrane protrusions, C, cell with clustered HER2 expression, F cell with flat cell surface and homogenous appearing HER2 expression. Note that brightness and contrast have been increased to visualize the HER2 expression on the flat regions thus overexposing the fluorescence of the surrounding cells.

Movie S4: Z-Stack acquisition of SKBR3 cells with EGFR enriched phenotype and clustered regions. Eight image Z-Stack (step size 2.063 µm) of dual labeled SKBR3 cells grown on a microchip. From left to right: HER2-QD565, EGFR-QD655, DIC, Merge (yellow HER2-QD565, cyan EGFR-QD655). Scale bar 20 µm. C = Cells with clustered HER2 expression pattern (bulk or EGFR enriched), LMPs = regions with large membrane protrusions.

Supplementary References

1. Peckys, D.B.; de Jonge, N. Studying the Stoichiometry of Epidermal Growth Factor Receptor in Intact Cells using Correlative Microscopy. *J Vis Exp* **2015**, 10.3791/53186, e53186, doi:10.3791/53186.

2. Peckys, D.B.; Baudoin, J.P.; Eder, M.; Werner, U.; de Jonge, N. Epidermal growth factor receptor subunit locations determined in hydrated cells with environmental scanning electron microscopy. *Sci Rep-Uk* **2013**, *3*, 2626, doi:10.1038/srep02626.

3. Peckys, D.B.; Korf, U.; de Jonge, N. Local variations of HER2 dimerization in breast cancer cells discovered by correlative fluorescence and liquid electron microscopy. *Sci Adv* **2015**, *1*, e1500165, doi:10.1126/sciadv.1500165.

4. Peckys, D.B.; Korf, U.; Wiemann, S.; de Jonge, N. Liquid-phase electron microscopy of molecular drug response in breast cancer cells reveals irresponsive cell subpopulations related to lack of HER2 homodimers. *Mol Biol Cell* **2017**, *28*, 3193-3202, doi:10.1091/mbc.E17-06-0381.
